# The role of male hypogonadism, aging, and chronic diseases in characterizing adult and elderly men with erectile dysfunction: a cross-sectional study

**DOI:** 10.1186/s12610-022-00182-8

**Published:** 2023-04-06

**Authors:** Giuseppe Lisco, Vincenzo Triggiani, Nicola Bartolomeo, Maria Isabella Ramunni, Carla Pelusi, Giovanni De Pergola, Edoardo Guastamacchia, Emilio Jirillo, Vito Angelo Giagulli

**Affiliations:** 1 Interdisciplinary Department of Medicine-Section of Internal Medicine, Geriatrics, Endocrinology and Rare Diseases, School of Medicine, University of Bari “Aldo Moro”, Piazza Giulio Cesare 11, 70124 Policlinico, Bari, Italy; 2 Outpatients Clinic of Endocrinology and Metabolic Disease, Conversano Hospital, Conversano, Bari, Italy; 3 Division of Endocrinology and Diabetes Prevention and Care, Department of Medical and Surgical Sciences (DIMEC), IRCCS Azienda Ospedaliero-Universitaria di Bologna, Alma Mater Studiorum University of Bologna, Bologna, Italy; 4 Research Hospital National Institute of Gastroenterology Saverio de Bellis, Castellana Grotte, Bari, Italy

**Keywords:** Erectile dysfunction, Testosterone, Charlson comorbidity index, Non-communicable chronic diseases, Male hypogonadism

## Abstract

**Background:**

Erectile function depends on a complex interaction between demographic, metabolic, vascular, hormonal, and psychological factors that trigger erectile dysfunction (ED). In the present study we carried out a cross-sectional study assessing the impact of non-communicable chronic diseases (NCDs), male hypogonadism, and demographic factors in characterizing men with ED. Four hundred thirty-three consecutive outpatients with ED were extracted from the electronic database from January 2017 to December 2019. The International Index of Erectile Function (IIEF) 5 score was used to diagnose ED and stratify its severity, standardized values of serum testosterone (10.5 nM/L) and luteinizing hormone (LH 9.4 IU/L) to diagnose and classify male hypogonadism and the Charlson Comorbidity Index (CCI) to weigh the role of each NCD on ED.

**Results:**

Forty-six percent of participants were eugonadal (EuG), 13% had organic hypogonadism (OrH), and the remaining 41% had functional hypogonadism (FuH). Hypogonadal men had a significantly lower IIEF 5 score (*p* < .0001) than EuG. FuH had a higher CCI than OrH and EuG (all *p* < .0001). In a multivariable model, only free T (FT) and Sex Hormone Binding Globulin (SHBG) showed a direct correlation with the IIEF 5 score (all *p* < .0001). Age and CCI had an inverse correlation with IIEF 5 score (all *p* < .0001).

**Conclusion:**

Serum FT, SHBG, and CCI are the leading determinants of ED severity. Besides overt hypogonadism, a relevant burden of severe NTCDs in middle-aged or older adults features the patient’s characteristics who will suffer from severe ED. Appropriate clinical approaches and, when necessary, treatments are required in these clusters of patients.

## Résumé

**Contexte:**

La fonction érectile dépend d’une interaction complexe entre les facteurs démographiques, métaboliques, vasculaires, hormonaux et psychologiques qui déclenchent la dysfonction érectile (DE). Dans la présente étude, nous avons mené ici une étude transversale évaluant l’impact des maladies chroniques non transmissibles (MNT), de l’hypogonadisme masculin et des facteurs démographiques dans la caractérisation des hommes atteints de dysfonction érectile. Quatre cent trente-trois patients externes consécutifs présentant une dysfonction érectile ont été extraits de la base de données électronique de janvier 2017 à décembre 2019. Le score de l’indice international de la fonction érectile (IIEF) 5 a été utilisé pour diagnostiquer la dysfonction érectile et stratifier sa gravité, les valeurs normalisées de la testostérone sérique (10,5 nM/L) et de l’hormone lutéinisante (LH 9,4 UI/L) pour diagnostiquer et classer l’hypogonadisme masculin, et l’indice de comorbidité de Charlson (ICC) pour évaluer le rôle de chaque MNT sur la DE.

**Résultats:**

Quarante-six pour cent des participants étaient eugonadiques (EuG), 13% avaient un hypogonadisme organique (OrH) et les 41% restants avaient un hypogonadisme fonctionnel (FuH). Les hommes hypogonadiques avaient un score IIEF 5 significativement plus faible (p < 0,0001) que EuG. Les hommes FuH avait un ICC plus élevé que les hommes OrH et EuG (tous p < .0001). Dans un modèle multivariable, seules la T libre (TL) et la globuline liant les hormones sexuelles (SHBG) ont montré une corrélation directe avec le score IIEF 5 (tous p <,0001). L’âge et l’ICC avaient une corrélation inverse avec le score IIEF 5 (tous p < 0,0001).

**Conclusion:**

La TL sérique, la SHBG et le CCI sont les principaux déterminants de la gravité de la DE. Outre l’hypogonadisme manifeste, une charge significative de MNT sévères chez les adultes d’âge moyen ou plus âgés dessine les caractéristiques du patient qui souffrira de DE sévère. Des approches cliniques appropriées et, si nécessaire, des traitements sont requis chez ces patients.

**Mots-clés:**

Dysfonction érectile, Testostérone, Indice de Comorbidité de Charlson, Maladies chroniques non transmissibles, Hypogonadisme masculin

## Background

Erectile dysfunction (ED) is a sexual disorder characterized by failure to obtain and maintain a penile erection sufficient to permit satisfactory sexual intercourse [1].

It is a common clinical condition over 40 years of age, with the prevalence increasing along with aging and chronic comorbidities (ranging from 10% in men > 40 years old to more than 50% in those over 70) [2, 3]. Given this assumption, the prevalence of ED is expected to increase over time due to the increase in life expectancy with an overall rise of men with non-communicable chronic diseases (NCDs) that could be considered ED risk factors [3–9].

ED strongly predicts poor health, worse quality of life, and increased mortality [10, 11]. In turn, ED is a risk factor for cardiovascular diseases (CVD) and all-cause mortality, as observed after comparing epidemiological data of sex- and age-matched individuals without ED. The burdens are more evident in younger than older men [4, 5]. Screening programs to identify and treat CVD or background CV risk in men complaining of ED is cost-effective [6, 7].

ED is a specific symptom of adult and age-related male hypogonadism [8]. Low circulating levels of testosterone induce ED, and this relation is also maintained in men with NCDs in which the severity of background health condition appears strictly related to the seriousness of sexual dysfunctions [9]. Low sexual drive can be related to low serum testosterone. It could be improved by non-hormonal (testosterone) therapy when ED is attributable to dysmetabolic and non-organic causes [12–14]. Low circulating testosterone and ED independently predict early death, as pointed out by the European Male Ageing Study [15]. Guidelines from the British Society for Sexual Medicine suggest measuring serum testosterone levels in all patients complaining of ED [16].

Male hypogonadism occurs when unequivocally low morning testosterone levels are detected. Male hypogonadism results from a primitive testicle deficiency (primary hypogonadism) and hypothalamic-pituitary injury or dysfunction (secondary hypogonadism). Both depend on organic (congenital or acquired) or functional causes. Acquired male hypogonadism in middle-aged or older adults with NCDs is defined as functional hypogonadism. Conversely, organic forms of male hypogonadism include every condition in which an exogen injury or endogen cause affects the hypothalamic-pituitary-testicular axis irreversibly [17].

Testosterone replacement therapy (TRT) is necessary when unequivocally and considerably low testosterone levels occur, especially in the case of organic hypogonadism [17]. In patients with near-normal or moderately low testosterone levels, such as men with NCDs and secondary hypogonadism, the benefit-to-risk ratio of TRT is still partially unknown [18]. Alternative treatments to restore T levels include medical and non-medical management of the leading causes of each chronic disease [17, 19, 20]. TRT improves ED more in individuals with severe hypogonadism, such as those having serum testosterone < 230 ng/dL (< 8 nM/L), than those with mild or moderate NCD-induced hypogonadism (i.e., dysmetabolic conditions) [20, 21]. Therefore, sexual dysfunction in hypogonadal men with NCDs could be improved by other specific therapies [20–24], lifestyle changes, bariatric surgery, or treatment of sleep disorders [21, 23–25].

The measurement of serum testosterone is also essential when symptomatic therapies (e.g., phosphodiesterase 5 inhibitors or low-energy shock-wave therapy) fail to improve ED significantly [26, 27].

In this cross-sectional study, we analyzed the role of male hypogonadism (expressed as low levels of serum testosterone), advanced age, and the number and severity of chronic comorbidities in contributing to ED. The aim was to define how these variables could be useful in clustering men with ED to reach personalized clinical outcomes and improve the appropriateness of medical and non-medical management of ED.

## Materials and methods

### Study design and institution

It was a cross-sectional study encompassing data from three years (January 2017 - December 2019) from a secondary center for endocrine and metabolic care, namely the” Outpatients Clinic of Endocrinology and Metabolic Disease of Conversano Hospital” (Conversano, Bari, Italy). All procedures were carried out according to the ethical principles inspired by good clinical practice and the Declaration of Helsinki.

The study was approved by the Ethical Committee of the University of Bari (protocol number 6454, July 2020) and the Azienda Sanitaria Locale (protocol number 1294, October 2020). Patients who signed informed consent to treat their data and provide personal information for clinical research purposes were included in our analyses. Patients denying informed consent to treat their clinical, laboratory, and other personal data are automatically excluded from the analyses. This approach is part of our clinical practice and is carried out systematically during each first visit.

Anamnestic data, clinical symptoms and signs, and laboratory data were searched in the institutional database and analyzed after Ethics Committee approval. The study aimed to assess the impact of overt hypogonadism (serum testosterone < 10.5 nM/l or 300 ng/dL) [17, 19, 23] and chronic comorbidities in classifying men with ED.

### Diagnostic workup

The initial workup had included the following steps: (a) collection of detailed medical, pharmacological, and sexual history; (b) check for smoking status; (c) physical examination and anthropometry (office blood pressure, body weight, height, body mass index, and waist circumference); (d) self-administrable questionnaire (i.e., the International Index of Erectile Function 5-item or IIEF 5) to diagnose and classify the severity of ED [28]; (e) laboratory tests, including fasting plasma glucose, glycated hemoglobin (HbA1c), lipid profile, total serum testosterone, sex hormone-binding globulin (SHBG), and gonadotropins (LH, FSH). Other laboratory measurements (e.g., fasting serum prolactin) and additional investigations (e.g., a workup for obstructive sleep apnea) were provided to individuals with specific signs and symptoms.

Blood specimens were collected from 8:00 to 9:00 am, after overnight fasting, and stored at -20 °C until analyzed. Two consecutive venous samples were collected from each patient in the morning (08:00–09:00 am), two weeks apart, to assess serum total testosterone and SHBG levels. Samples were analyzed in our laboratory as per habitual practice through standardized methods since 2006 [29]. Serum LH, FSH, total serum testosterone, and SHBG were measured by commercial immunometric assays (Immulite, EURO/DPC, UK), while serum-free testosterone (FT) was calculated by the Vermeulen’s formula [30]. Reference range, obtained from adult normal-weighted fertile men (41.5 ± 3.1 years), were the following: 7.5 ± 2.6 IU/L, 6.6 ± 2.5 IU/L, 450 ± 90 ng/dL (15.6 ± 3.1 nM/L), 45.4 ± 5.1nM/L, and 12 ± 2.2 ng/dL for LH, FSH, total serum testosterone, SHBG, and FT respectively. Intra- and inter-assay coefficients of variation of these methods were < 8% and < 10%, respectively [14, 18]. Serum testosterone and SHBG values were calculated as the mean of two consecutive measurements.

ED was diagnosed and staged (severity) according to the IIEF5 score. Men with scores < 22 were diagnosed with ED, and ED was classified as mild for scores 17–21, mild to moderate for scores 12–16, moderate for scores 8–11, and severe for scores 5–7 [28]. Self-reported or newly diagnosed comorbidities were used to calculate the Charlson Comorbidity Index (CCI) [31, 32]. One point was assigned to each pre-existing condition: cerebrovascular diseases, chronic lung diseases, heart failure, connective tissue diseases, dementia, diabetes mellitus, mild liver disease, myocardial infarction, peripheral vascular disease, and ulcers. Any malignancy, diabetes mellitus with end-organ damage, hemiplegia, leukemia, lymphoma, and moderate-to-severe chronic kidney disease (i.e., eGFR < 60 ml/min) were assigned 2 points each. We posted moderate or severe liver diseases 3 points each, and 6 points in the case of acquired immunodeficiency syndrome and solid metastatic tumor [31].

Since the CCI does not include well-known risk factors of ED, such as arterial hypertension, dyslipidemia, obstructive sleep apnea syndrome, and tobacco smoking [2–5], we developed a modified CCI (mCCI) to include the abovementioned variables assigning 1 point each [6, 33].

### Study participants

**Fig. 1:**
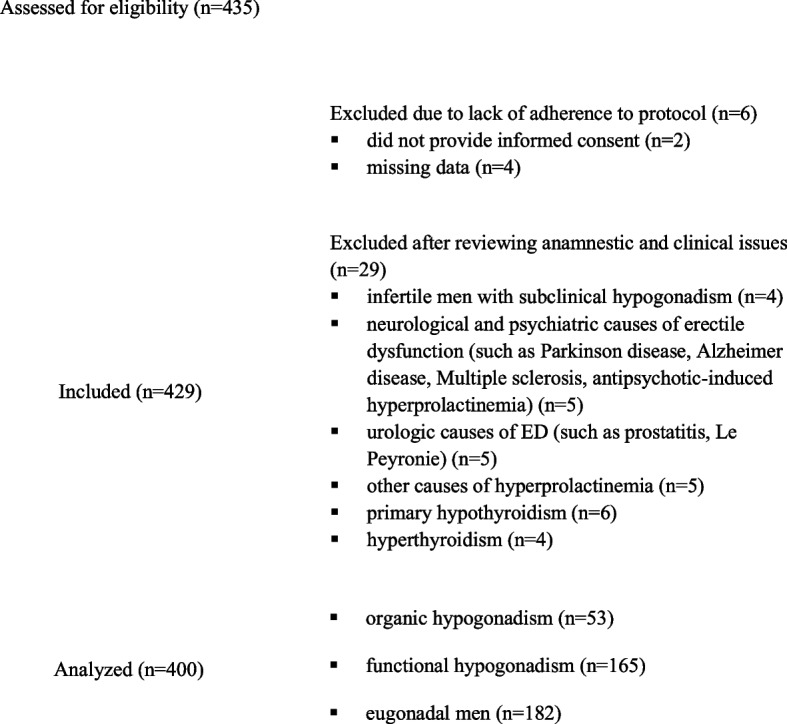
Selection of study participants according to the study protocol

Data from 435 Italian patients admitted to our Outpatient Unit due to ED, were collected and analyzed (Fig. 1). Among them, data from six were excluded due to the following causes: two did not provide informed consent and miss of interest data in four. Further, 29 patients were excluded after reviewing specific anamnestic, clinic, and therapeutic issues: four had idiopathic infertility and subclinical (serum testosterone levels ≥ 300ng/dL and LH ≥ 9.4 IU/L) primary hypogonadism (two individuals were diagnosed with oligoasthenospermia and overweight; the other two had oligospermia and the metabolic syndrome), five had psychiatric comorbidities, five had relevant urological disorders, five had hyperprolactinemia (defined as confirmed prolactin levels over 35 ng/mL) [34], six had overt hypothyroidism (Thyroid-Stimulating Hormone or TSH above the upper limit of the reference range and FT4 < 11.5 pM/L), and four had subclinical (TSH < 0.35 mU/L and FT4 ≤ 23 pM/L) and overt (TSH < 0.35 mU/L and FT4 > 23 pM/L) hyperthyroidism [35].

Laboratory criteria to classify gonadal status were according to standard recommendations [36]. Eugonadism (EuG) was defined in the case of total serum testosterone ≥ 300 ng/dL and LH ≤ 9.4 IU/L. Secondary hypogonadism was defined as serum testosterone ≤ 300 ng/dL and LH ≤ 9.4 IU/L. Primary hypogonadism was defined as testosterone ≤ 300 ng/dL and LH ≥ 9.4 IU/L [17, 19, 23, 37]. Hypogonadal patients were classified as having either “organic hypogonadism” (OrH) or “functional hypogonadism” (FuH) [12]. OrH included participants with a clinical history of congenital and acquired hypogonadism exhibiting unequivocally eunuchoid aspect, specific signs of hypogonadism, and those with serum testosterone levels < 200 ng/dL (6.9 nM/L). Patients with less specific symptoms and signs (e.g., low energy, depressed mood, loss of body hair), NCDs, without evidence of hypothalamus-pituitary-testes axis disruption, and serum testosterone levels between 200 and 300 ng/dL were reasonably deemed as having FuH.

### Outcomes

The primary study outcome was to cross-sectionally evaluate any differences in background characteristics and ED severity of the study population. Patients were classified as OrH (total serum testosterone < 200 ng/dL or unequivocal clinical history of organic hypogonadism), FuH (serum total testosterone 200–300 ng/dL and less specific signs and symptoms of hypogonadism without a personal history of organic hypogonadism), and EuG (total serum testosterone > 300 ng/dL).

The secondary outcome was to analyze the relationship between the IIEF 5 score and anthropometric and clinical characteristics (age; body mass index or BMI; CCI; mCCI), laboratory parameters (follicle-stimulating hormone or FSH, luteinizing-hormone or LH, SHBG, and FT) in all subgroups.

### Statistical analysis

Shapiro-Wilk’s statistics were used to test normality for continuous variables, and an appropriate function was applied to transform those not showing a normal distribution. All variables and their possible transformations have skewed data distributions and are therefore expressed as median and interquartile. The Kruskal Wallis test for non-parametric analysis of variance was used to analyze the differences between OrH, FuH, and EuG. Pairwise multiple comparisons were adjusted according to the Dunn correction.

The distribution of patients in each ED category, age, CCI, and mCCI were described as frequency and proportion. When necessary, the Chi-square test or the Fisher Exact test were employed to test their associations with OrH, FuH, and EuG. Pairwise multiple comparisons between proportions were adjusted according to the Tukey correction.

Univariate and multivariable generalized linear models (GLM) were applied to evaluate the effect of some parameters (age, BMI, waist circumference or WC, CCI, mCCI, FSH, LH, SHBG, FT, eugonadal group, and hypogonadal groups) on the IIEF 5 score. The multivariate GLM model was assessed to evaluate which parameters could have an independent effect in increasing the IEFF 5 score. The model did not include the variable testosterone to avoid multicollinearity effects with FT. Multicollinearity between SHBG and FT was also investigated but not present, and both predictors were included in the analysis.

Using Akaike Information Criterion (AIC), a stepwise selection was used to estimate the final model. The results of the generalized linear model are expressed by the estimated effects B, standard errors, the standardized regression coefficients β to evaluate the effect of the predictor not in its unit of measurement, and the p values of the Student’s t-tests. For one-way ANOVA applied to Ft level with three means (2.4, 5.2, 6.8) using a two-sided significance level of 0.05, assuming an error standard deviation of 0.8 and group sample sizes of (53, 165, 182) yields a power of 1.0. In a multivariable regression model, for a Type III F test of one predictor adjusting for the other three predictors (excluding the intercept) with a significance level of 0.05, assuming a conditional model with fixed predictors and an R-square of 0.58 in the full model, a sample size of 400 has a power of 0.99 to detect an R-square difference of 0.03.

All tests of statistical significance were two-tailed, and *p*-values less than 0.05 were considered statistically significant. Statistical analysis was performed using the SAS/STAT® Statistics version 9.4 (SAS Institute, Cary, NC, USA).

## Results

### Characteristics of the study population

Patients’ characteristics and comparisons between the three groups are shown in Table 1. Fifty-three out of 400 (13.25%) participants had OrH (total testosterone: 120–170 ng/dL), and 165 (41.25%) had FuH (total testosterone: 243–288 ng/dL). One-hundred eighty-two patients (45.5%) were EuG (total serum testosterone: 320–390 ng/dL). The levels of FT overlapped those of total testosterone, thus confirming the diagnostic accuracy of the gonadal status in all [17]. Patients with OrH were significantly younger (31–54 years) than FuH and EuG men. Individuals with FuH were considerably older than EuG. Primary OrH had the following etiologies: Klinefelter syndrome in 4 (28.6%), primary hypogonadism with unknown causes in 4 (28.6%), bilateral orchiectomy due to primitive testicular cancer in 2 (14.3%), cryptorchidism in 3 (21.4%), and one had infective orchitis in the past. Nineteen-nine of 39 patients (48.7%) with secondary OrH had a previous pituitary surgery due to pituitary adenoma, 9 (23.1%) have isolated idiopathic hypogonadism hypogonadotropic, 4 (10.2%) were diagnosed with the Kallmann syndrome, 2 (5.1%) were diagnosed with infiltrative pituitary disease, and 5 (12.8%) had a brain trauma in the past.

**Table 1:** Characteristics (continuous parameters) of the study population. Comparison between the three groups (Organic Hypogonadal group, Functional Hypogonadal group, Eugonadal group)

Parameters	Organic hypogonadism *n* = 53	Functional hypogonadism *n* = 165	Eugonadism *n* = 182	*p* (K-W)	OrH vs. FuH	OrH vs. EuG	FuH vs. EuG
	Median [Interquartile Range]						
BMI, kg/m^2^	27 [24.0-33.3]	32.7 [28.6–37.0]	28.7 [25.6–31.9]	**< 0.001**	**< 0.001**	0.496	**< 0.001**
CCI, score	1 [0–2]	5 [3–6]	3 [2–4]	**< 0.001**	**< 0.001**	**< 0.001**	**< 0.001**
mCCI, score	3 [1–4]	7 [5–8]	5 [4–6]	**< 0.001**	**< 0.001**	**< 0.001**	**< 0.001**
WC, cm	103 [91–114.0]	111 [102–122]	101 [95–108]	**< 0.001**	**0.011**	0.719	**< 0.001**
Age, years	42 [31.3–54.0]	65 [56–72]	60.5 [52–66]	**< 0.001**	**< 0.001**	**< 0.001**	**< 0.001**
FSH, UI/L	3.1 [1.8–15.3]	7.7 [6.9–8.6]	7.3 [6.9–8.1]	**0.022**	0.259	0.415	0.076
FT, ng/dL	2.4 [1.8-3.0]	5.2 [4.8–5.7]	6.7 [6.2–7.3]	**< 0.001**	**< 0.001**	**< 0.001**	**< 0.001**
IIEF 5, score	12 [8–14]	10 [8–12]	14 [13–16]	**< 0.001**	0.212	**< 0.001**	**< 0.001**
LH, UI/L	2.3 [1.8–10.0]	6.7 [5.9–7.5]	6.4 [5.7–7.1]	**< 0.001**	**0.001**	**0.001**	0.190
SHBG, nM/L	38 [35–46]	33 [30–36]	37 [34–42]	**< 0.001**	**< 0.001**	0.451	**< 0.001**
T, ng/dL	150 [120–171]	266 [243.5–288]	356 [320–390]	**< 0.001**	**< 0.001**	**< 0.001**	**< 0.001**

*Abbreviations*: *OrH*  organic hypogonadism, *FuH*  functional hypogonadism, *EuG*  eugonadism, *BMI*  body mass index, *CCI*  Charlson Comorbidity Index, *mCCI*  modified Charlson Comorbidity Index, *WC*  waist circumference, *FSH*  follicle-stimulating hormone, *FT*  free testosterone, *IIEF 5*  International Index of Erectile Function 5 itmes;

*LH*  luteinizing-hormone, *SHBG*  sex hormone biding globulin, *T*  testosterone, *K-W*  Kruscal-Wallis Test

ED was more severe in men with OrH and FuH than EuG. A greater frequency of severe ED was found in men with FuH than OrH (16.4% vs. 13.2%). EuG individuals frequently had a mild-to-moderate ED. Anthropometric parameters differently characterized the three categories of participants. OrH and EuG were usually overweight, while those with FuH exhibited a central obesity pattern (waist circumference: 102–122 cm) [38]. Men with FuH had lower levels of SHBG compared to the remaining two subgroups. Serum LH levels were statistically lower in OrH than FuH and EuG, while circulating FSH levels were not statistically different among the three subgroups of patients. Among men with OrH, 14 (26.4%) had LH levels > 9.4 IU/L, and 39 (73,6%) had serum LH levels < 9.4 IU/L. Age, anthropometric parameters (BMI and WC), and hormone levels (testosterone, FT, and SHBG) were not statistically different between the two sub-groups of hypogonadism. Besides LH levels, serum FSH > 8 IU/L was significantly more frequent in primary than a secondary form of OrH (*p* < .0001) (data not shown).

### Characterizing comorbidities across all categories of participants with ED

All patients complained of ED. After the assessment, ED was diagnosed in all hypogonadal individuals and 93% of EuG. The CCI and mCCI scores were statistically higher in FuH and EuG (*p* < .0001), while patients with OrH had a statistically lower score than the latter two subgroups of participants (Table 1). Relevant clinical conditions such as arterial hypertension, diabetes mellitus with end-organ damage, and moderate or severe chronic kidney disease occurred more frequently in older men with FuH and EuG than in those with OrH (Table 2). Chronic heart failure, mild liver disease, and diabetic ulcers were more frequently observed in men with FuH than in EuG. Similarly, arterial hypertension, sleep apnea, and dyslipidemia were more prevalent in FuH than EuG. Diabetes mellitus without end-organ damage was more frequently found in EuG than FuH (Table 2).

**Table 2:** Characteristics (categorical parameters) of included subjects. Comparison between OrH, FuH, and EuG men

Parameter		Organic hypogonadism *n* = 53	Functional hypogonadism *n* = 165	Eugonadism *n* = 182	*p* (CSQ)	OrH vs. FuH	OrH vs. EuG	FuH vs. EuG
		n (%)						
ED (prevalence)		53 (100%)	165 (100%)	168 (92.3%)	**0.0002**	0.843	0.048	**< 0.001**
ED (severity)								
Severe		7 (13.2%)	27 (16.4%)	1 (0.6%)	**< 0.0001**	0.722	**< 0.001**	**< 0.001**
Moderate		19 (35.8%)	64 (38.8%)	15 (8.2%)				
Mild-to-moderate		25 (47.2%)	73 (44.2%)	123 (67.6%)				
Mild		2 (3.8%)	1 (0.6%)	29 (15.9%)				
Normal		0 (0%)	0 (0%)	14 (7.7%)				
Age classes, years								
< 50		36 (67.9%)	19 (11.9%)	32 (17.6%)	**< 0.0001**	**< 0.001**	**< 0.001**	**< 0.001**
50–59		11 (20.8%)	38 (23%)	49 (26.9%)				
60–69		5 (9.4%)	47 (28.5%)	75 (41.2%)				
70+		1 (1.9%)	61 (37%)	26 (14.3%)				
Comorbidities included in the CCI								
1 point	Cerebrovascular disease	0 (0%)	18 (10.9%)	11 (6%)	**0.0200**	-	-	-
	Chronic lung disease	3 (5.7%)	43 (26.1%)	37 (20.3%)	**0.0061**	**0.010**	**0.030**	0.347
	Congestive heart failure	2 (3.8%)	32 (19.4%)	12 (6.6%)	**0.0002**	**0.010**	0.703	**< 0.001**
	Connective tissue disease	1 (1.9%)	8 (4.9%)	11 (6%)	0.4707	-	-	-
	Dementia	0 (0%)	5 (3%)	5 (2.8%)	0.6143^a^	-	-	-
	Diabetes without end-organ damage	10 (18.9%)	47 (28.5%)	80 (44%)	**0.0004**	0.327	**< 0.001**	**< 0.001**
	Mild liver disease	21 (36.6%)	104 (63%)	72 (39.6%)	**< 0.0001**	**< 0.001**	0.990	**< 0.001**
	Myocardial infarction	0 (0%)	38 (23%)	29 (15.9%)	**0.0004**	**< 0.001**	**< 0.001**	0.227
	Peripheral vascular disease	0 (0%)	20 (12.1%)	16 (8.8%)	**0.0271**	**0.020**	**0.040**	0.594
	Ulcer	0 (0%)	28 (17%)	12 (6.6%)	**0.0002**	**< 0.001**	0.139	**< 0.001**
2 points	Any tumor	3 (5.7%)	21 (12.7%)	27 (14.8%)	0.2116	-	-	-
	Diabetes with end-organ damage	1 (1.9%)	91 (55.2%)	52 (28.6%)	**< 0.0001**	**< 0.001**	**< 0.001**	**< 0.001**
	Hemiplegia	0 (0%)	1 (0.6%)	1 (0.6%)	1.0000^a^	-	-	-
	Leukemia	0 (0%)	1 (0.6%)	3 (1.7%)	0.7879^a^	-	-	-
	Lymphoma	0 (0%)	1 (0.6%)	2 (1.1%)	1.0000^a^	-	-	-
	Moderate or severe kidney disease	0 (0%)	46 (27.9%)	20 (11.1%)	**< 0.0001**	**< 0.001**	**0.010**	**< 0.001**
3 points	Moderate or severe liver disease	2 (3.8%)	5 (3%)	6 (3.3%)	0.9643	-	-	-
6 points	AIDS	0	0	0	-			
	Metastatic solid tumor	1 (1.9%)	2 (1.2%)	5 (2.8%)	0.6200^a^	-	-	-
Other data								
	Arterial hypertension	22 (41.5%)	152 (92.1%)	145 (79.7%)	**< 0.0001**	**< 0.001**	**< 0.001**	**< 0.001**
	Dyslipidemia	36 (67.9%)	155 (93.9%)	156 (85.7%)	**< 0.0001**	**< 0.001**	**< 0.001**	**0.040**
	Current cigarette smokers	22 (41.5%)	72 (43.6%)	81 (44.5%)	0.9272	-	-	-
	Obstructive sleep apnea Syndrome	5 (9.4%)	55 (33.3%)	22 (12.2%)	**< 0.0001**	**< 0.001**	0.990	**< 0.001**

*Abbreviations*: *OrH*  organic hypogonadism, *FuH*  functional hypogonadism, *EuG*  eugonadism, *ED*  erectile dysfunction, *CCI*  Charlson Comorbidity Index, *AIDS*  acquired immunodeficiency syndrome, *CSQ*  Chi-Square Test,

a = “Fisher Exact Test”

### Characterizing the effects of anthropometric characteristics, clinical conditions, and hormonal parameters on IIEF 5 scores across all categories of participants

The univariate and multivariable generalized linear models were used to evaluate the effect of anthropometric characteristics, clinical comorbidities (expressed as CCI and mCCI), hormonal parameters (FSH, LH, SHBG, and FT), and hypogonadism on the IIEF 5 score. In univariate analyses, age, BMI, mCCI score, and hypogonadism were negatively correlated (*p* < .0001), while SHBG and FT were positively correlated with the IIEF 5 score (Table 3). The multivariable analysis showed that age and mCCI provided the best contribution (with inverse correlation) to the IIEF 5 score (*p* < .0001). SHBG and FT levels had a positive correlation with the IIEF 5 score (*p* < .0001). The coefficient β showed that FT levels had the greatest (negative) correlation with the IIEF 5 score, followed by the mCCI score (Table 3).

**Table 3:** Results of the generalized linear model applied to the relationship between the International Index of Erectile Function 5-item and the parameters Age, body mass index, modified Charlson Comorbidity Index, Follicle-Stimulating hormone, Luteinizing-Hormone, Sex-Hormone Binding Globulin, Free Testosterone, and the gonadal status defined as a binomial variable (hypogonadism, if total Testosterone lower than 300 ng/dL; eugonadism, if total Testosterone equal to or more than 300 ng/dL)

Parameter	Univariate		Multivariable		
	B (SE)	*p* value	B (SE)	β	*p* value
Age	-0.06 (0.01)	< 0.0001	-0.05 (0.01)	-0.16	**< 0.0001**
BMI	-0.24 (0.03)	< 0.0001	-	-	-
mCCI	-0.62 (0.07)	< 0.0001	-0.34 (0.06)	-0.21	**< 0.0001**
FSH	+ 0.02 (0.03)	0.6314	-	-	-
LH	-0.004 (0.073)	0.9574	-	-	-
SHBG	+ 0.29 (0.02)	< 0.0001	+ 0.28 (0.02)	+ 0.49	**< 0.0001**
FT	+ 0.99 (0.11)	< 0.0001	+ 1.35 (0.08)	+ 0.55	**< 0.0001**
Gonadal status					
OrH vs. EuG	-3.71 (0.54)	< 0.0001	-	-	-
FuH vs. EuG	-4.63 (0.37)	< 0.0001	-	-	-

*Abbreviations* *BMI*  body mass index, *mCCI*  modified Charlson Comorbidity Index, *FSH*  follicle-stimulating hormone, *LH*  luteinizing-hormone, *SHBG*  sex hormone biding globulin, *FT*  free testosterone; *OrH*  organic hypogonadism, *FuH*  functional hypogonadism, *EuG*  eugonadism.

## Discussion

Our results provide information about the clinical characteristics of outpatients complaining of ED. The study results highlighted a high prevalence of EuG men, suggesting that around half of them had normal background serum testosterone levels. As expected, a more significant proportion of men with OrH and FuH than EuG was diagnosed with ED, being ED more severe in FuH than OrH. The findings are consistent with the fact that the frequency and severity of ED are driven by low serum testosterone concentration (male hypogonadism vs. eugonadal) and burdens related to chronic background comorbidities (ED more severe in FuH than EuG). Age and mCCI were strictly associated (inverse relation) with the severity of ED, and SHBG and FT levels positively related to it. These findings provide a better understanding of background characterization of the study population, representative of men complaining of ED who were referred to a specialized center for appropriate management.

An intricate interplay between psychological, neurological, endocrine, and vascular characteristics regulates erectile function. ED increases stress, worries, anxiety, and frustration leading to worse quality of life [39]. On the other hand, ED may be a clinical manifestation of cardiovascular background disease, especially in young. Moreover, aging, unhealthy lifestyles (i.e., smoking, alcohol abuse, physical inactivity, unhealthy eating), hypogonadism, and NCDs (cardiovascular, metabolic, respiratory, malignancies, and neuropsychiatric diseases) negatively affect erectile performance [2]. According to this reverse perspective, ED may indicate poor health, reduced quality of life, increased risk of future CVD, and mortality [4, 5].

There is a real need to precociously diagnose ED and its background etiology to provide more appropriate treatments and “patient-tailored” management. Besides symptomatic treatment aimed to improve erectile performance, proper therapeutic goals move toward a curative approach by identifying triggering conditions associated with ED [23, 40]. For example, phosphodiesterase type 5 inhibitors (PDE5i) are considered the first-line therapy for ED [6]. Treatment with PDE5i fails in around 40% of patients, especially in men with type 2 diabetes [16]. Similarly, TRT is less effective when ED is not attributable to moderate or severe hypogonadism [21].

In this cross-sectional study, we used standardized and validated methods for characterizing patients complaining of ED, including the IIEF 5 score to diagnose ED and classify its severity [28], testosterone cut-offs to identify and classify hypogonadal men as the EMAS study suggested [36], the CCI and mCCI to summarize the overall burdens related to aging, number, and severity of chronic comorbidities [31, 32]. More than half participants (54.5%) were diagnosed with hypogonadism (testosterone ≤ 300 ng/dL): 13.25% with OrH (both pre-pubertal or post-pubertal) and 41.25% with FuH. These data are similar to those reported by Corona et al. [41], and slightly higher than those found in another observation among healthy age-matched European men [36]. Serum total testosterone thresholds in defining hypogonadism are generally accepted [17, 18, 33, 37]. Conversely, more controversy exists in recommending specific cut-offs for distinguishing OrH and FuH (mostly adult and older men). The Endocrine Society considers an endogenous total testosterone concentration of 5.2 nM/L (150 ng/dL) as a highly predictive threshold for suspecting an organic etiology of male hypogonadism. Other authors put forward the threshold to 6.1 nM/L (175 ng/dL) [42]. By following the Endocrine society guideline, we distinguished OrH from FuH, as suggested by previous studies [19, 43]. None with FuH had plasma LH levels above the threshold of 9.4 IU/L, indicating that all subjects with FuH were affected by secondary hypogonadism. Conversely, 26% of men with OrH had serum LH > 9.4 IU/L, suggesting they had primary hypogonadism. Interestingly, we found that OrH prevalence in our series (3.5%) was higher compared to that observed in the European Male Aging (EMAS) study (2%) [36]. Hence, these data point out the central role of hypogonadism in men with ED [21]. Testosterone has a crucial role in driving sexual behavior and maintaining erectile function by upregulating the NO synthase activity at the endothelial site (eNOS) and in non-adrenergic, non-cholinergic neurons (nNOS) and by downregulating RhoA-ROCK pathway [21]. Testosterone (especially FT) could be considered a link between hypogonadism, cardio-metabolic parameters, ED, and mortality in men [36].

To our knowledge, a standardized cut-off value of FSH to differentiate primary and secondary OrH currently needs to be improved. In our study, by applying the standard threshold of plasma LH levels (9.4 IU/L), we found that serum FSH equal to 8 IU/L could reinforce clinical decisions in distinguishing primary and secondary OrH. This finding is supported by a few data currently available in the literature suggesting that a confirmed plasma FSH > 8 IU/L is usually associated with irreversible testicular damage, hence defining primary hypogonadism [44–48]. In fact, the Italian Agency for Drug Administration allows the reimbursement of FSH replacement therapy only in men with idiopathic impaired spermatogenesis having confirmed serum FSH levels less than 8 IU/L (http://www.agenziafarmaco.gov.it/).

Serum SHBG levels may be affected by age, hormones (i.e., thyroid hormones, estrogens, androgens), certain medications, and NCDs [49]. In this study, men with FuH had statistically lower SHBG levels than OrH and EuG. Since FuH had higher CCI scores than OrH and EuG, endogenous SHBG levels can be considered an additional marker of poor health. It is particularly true for middle-aged and older men with ED, since it is well-known that serum SHBG levels increase with aging in healthy men [49]. In our series of participants, serum FT trended in parallel with testosterone levels. In EuG, we found that a mean of 6.7 ng/dL does not deviate by much from the one proposed by others [50], further indicating the accuracy of the subdivision of our series of ED men.

Our study pointed out that hypogonadal young men (< 50 years old) complaining of ED may probably have an OrH, while middle-aged and elderly hypogonadal men (> 50 years old) are likely to be FuH. Besides age, being overweight and obese can induce ED [51], with testosterone deficiency playing a pathophysiological role in this group of patients (Table 1). ED is also observed in metabolically healthy obese men, highlighting the involvement of several pathophysiological mechanisms, such as endothelial dysfunction, physical inactivity, and psychological factors. Our findings support the screening of ED among obese individuals, regardless of the suspicion of overt hypogonadism (Table 1) [52, 53].

Clinical features of hypogonadism in adult and older men may often overlap the epidemiology of NCDs [9]. TRT in hypogonadal men could not improve well-being and sexual function whenever eugonadal status could have been re-established [53]. This finding underlines the role of additional factors other than hypogonadism in determining sexual dysfunction. These factors include well-recognized risk factors for NCDs, such as cigarette smoke, physical inactivity, alcohol abuse, and unhealthy eating [2].

However, both subgroups of hypogonadal men had more severe ED than EuG. In addition, men with FuH had statistically higher values of CCI and mCCI, and men with OrH also had worse IIEF 5 scores than EuG, all affected by moderate to severe ED (Table 2). Interestingly, less than 8% of EuG patients had a normal IIEF 5 score. These eugonadal men were younger (< 55 years) and had the CCI and mCCI below their means of 3 and 5, respectively. This cluster of patients had the so-called “subclinical ED” generated by a negative loop between psychological, relational factors, and organic determinants facilitating sexual dysfunction in relatively healthy men (i.e., metabolic healthy obese men) [53, 54]. Given that men with EuG were younger and had fewer organic determinants of ED than we speculate that the natural clinical history of chronic diseases could deteriorate the hypothalamus-pituitary testicular axis [55]. The persistence of overt male hypogonadism may worsen the general health and ED severity [56]. On the other hand, men with OrH and lower serum testosterone levels than FuH showed a higher prevalence of metabolic disturbances (i.e., type 2 diabetes mellitus) than the general Italian population [57]. ED links hypogonadism to generally poor health and reduced quality of life, thus indicating a risk factor for all-cause mortality in adult men [4, 5, 15].

Recently, other authors have evaluated a different tool (a pharmaceutical-based instrument) weighing the possible relationship between multimorbidity, serum testosterone levels, and ED [9]. Tools to assess the relationship between the burden of NCDs and different outcomes are not comparable [58]. We first used the CCI only, then modified the classical score for a second look since it did not include essential trigger factors for ED, such as arterial hypertension, sleep apnea, and dyslipidemia (mCCI) [5, 6]. Moreover, both tool did not consider pharmacological therapy. Chronic assumption of some medications can be associated with ED [59, 60]. In our study, FuH and EuG middle-aged and older men were on antiandrogens therapy (i.e., spironolactone), 5-alfa reductase inhibitors, and antidepressants in only 0.5%, 0.7%, and 1.5%, respectively. Around 8% of patients declared to have sporadically assumed PDE5i for a brief period, and approximately 15% of OrH were on TRT. TRT and PDE5i were discontinued at least three weeks before laboratory and clinical evaluations to provide an appropriate washout of confounding factors. Conversely, 82% of both FuH and EuG men and 38% of OrH were taking antihypertensive medications, including angiotensin-converting enzyme inhibitors (61%), angiotensin receptor blockers (15%), calcium channel blockers (23%), thiazide diuretics (18%), and β-blockers (9%) that may play an overall neutral effect on ED [61]. Around 70% of EuG and FuH and 14% of OrH men were on anti-hyperglycemic medications (e.g., dulaglutide, liraglutide, metformin, and empagliflozin) that may improve the erectile function, as studies suggested [19, 62–65]. Overall, the CCI and mCCI can effectively recognize and synthetize the burden of chronic diseases when assessing men with ED.

Age, BMI, mCCI, and hypogonadism negatively affected erectile function in men. However, those men with higher FT and SHBG levels had a higher IIEF 5 score, highlighting the negative role of aging, obesity, and chronic diseases on erectile function. Serum FT and SHBG could be biomarkers of erectile function in middle-aged and older men. This data indicated that circulating levels of FT are more accurate than serum testosterone alone in identifying patients with ED, as recently pointed out [66, 67]. The diagnosis, management, and follow-up of patients with ED often appear relatively complex. This study provided valuable information to clinicians for a better characterization of men with ED, considering hypogonadism (organic or functional), risk factors for ED, and, above all, the severity of NCDs often present with functional hypogonadism.

Our study has some strengths and limitations that should be addressed. The cross-sectional nature of the study can be the most significant limitation, and thus it cannot explain per se a cause-effect relationship between the variable we analyzed. Moreover, our results came from a cohort of men complaining of ED, and they could be different compared to the general population or patients referring from various settings (i.e., urologists). On the other hand, well-standardized clinical, instrumental, and laboratory assessments and the consecutive selection of patients who met specific criteria reduced the risk of selection bias in terms of diagnostic criteria, clinical judgment, and overall management. All in all, two are the main strengths of our study: first, it was conducted in a real-life setting and included a good number of men; second, selection criteria were rigorously based on the interpretation of hormonal parameters, diagnostic criteria for male hypogonadism [17, 18, 33, 36], ED [32], and NCDs [28].

## Conclusion

Our data suggest that serum FT, SHBG, and mCCI are the leading determinants of ED severity. The concomitant presence of overt hypogonadism and multiple chronic comorbidities in middle-aged and older adults features the characteristics of men with moderate or severe ED. Clinicians are requested to characterize patients complaining of ED methodically and improve the appropriateness of clinical management. However, prospective and long-term controlled interventional trials are needed to better understand the mechanism between different clinical and hormonal determinants and ED, even considering ED is a symptom of NCDs.

## Data Availability

All data were collected in computerized medical records as part of routine practice. The datasets generated and analyzed during the current study are available from the corresponding author on reasonable request.

## Abbreviations

AIC: Akaike Information Criterion
BMI: Body Mass Index
CVD: Cardiovascular Diseases
CCI: Charlson Comorbidity Index
ED: Erectile Dysfunction
eGFR: estimated Glomerular Filtration Rate
EMAS: (The) European Male Aging Study
eNOS: endothelial Nitric Oxide Synthase
EuG: Eugonadism
FSH: Follicle-Stimulating Hormone
FT: Free Testosterone
FT4: free thyroxine
GLM: Generalized Linear Models
FuH: Functional Hypogonadism
HbA1c: Glycated Hemoglobin
IIEF 5: International Index of Erectile Function 5
LH: Luteinizing-Hormone
mCCI: Modified Charlson Comorbidity Index
NCDs: Non-communicable Chronic Diseases
nNOS: neuronal Nitric Oxide Synthase
OrH: Organic Hypogonadism
PDE5i: PhosphodDesterase type 5 inhibitors
SHBG: Sex Hormone Binding Globulin
TRT: Testosterone Replacement Therapy
TSH: Thyroid-Stimulating Hormone
